# Design and bioinformatics analysis of genome-wide CLIP experiments

**DOI:** 10.1093/nar/gkv439

**Published:** 2015-05-09

**Authors:** Tao Wang, Guanghua Xiao, Yongjun Chu, Michael Q. Zhang, David R. Corey, Yang Xie

**Affiliations:** 1 Quantitative Biomedical Research Center, University of Texas Southwestern Medical Center, 5323 Harry Hines Boulevard, Dallas, TX 75390, USA; 2 Departments of Pharmacology and Biochemistry, University of Texas Southwestern Medical Center, 5323 Harry Hines Boulevard, Dallas, TX 75390, USA; 3 Department of Biological Sciences, Center for Systems Biology, The University of Texas at Dallas, Richardson, TX 75080, USA; 4 Bioinformatics Division, Center for Synthetic and System Biology, TNLIST, Tsinghua University, Beijing 100084, China; 5 Harold C. Simmons Comprehensive Cancer Center, University of Texas Southwestern Medical Center, 5323 Harry Hines Boulevard, Dallas, TX 75390, USA

## Abstract

The past decades have witnessed a surge of discoveries revealing RNA regulation as a central player in cellular processes. RNAs are regulated by RNA-binding proteins (RBPs) at all post-transcriptional stages, including splicing, transportation, stabilization and translation. Defects in the functions of these RBPs underlie a broad spectrum of human pathologies. Systematic identification of RBP functional targets is among the key biomedical research questions and provides a new direction for drug discovery. The advent of cross-linking immunoprecipitation coupled with high-throughput sequencing (genome-wide CLIP) technology has recently enabled the investigation of genome-wide RBP–RNA binding at single base-pair resolution. This technology has evolved through the development of three distinct versions: HITS-CLIP, PAR-CLIP and iCLIP. Meanwhile, numerous bioinformatics pipelines for handling the genome-wide CLIP data have also been developed. In this review, we discuss the genome-wide CLIP technology and focus on bioinformatics analysis. Specifically, we compare the strengths and weaknesses, as well as the scopes, of various bioinformatics tools. To assist readers in choosing optimal procedures for their analysis, we also review experimental design and procedures that affect bioinformatics analyses.

## INTRODUCTION

The diversity of RNA in sequence and structure underpins much of cell heterogeneity and complexity. RNA-binding proteins (RBPs) are proteins that bind to double- or single-stranded RNAs in cells and form ribonucleoprotein complexes with the bound RNAs. Located in either the nucleus or cytoplasm, or both, they engage in every step of the post-transcriptional modification process, including alternative splicing, regulation of mRNA levels, transport between cellular compartments, alternative polyadenylation, transcript stability, etc. (1,2). For example, the TIAR protein has been shown to be transported from the nucleus to the cytoplasm during Fas-mediated apoptotic cell death (3). One example of an intra-nuclear RBP is Yra1p, which has been found to be involved in mRNA export (4). Cytoplasmic RBPs, on the other hand, include Unr, which has been shown to be required for internally initiating the translation of human rhinovirus RNA (5).

RBPs bind target RNAs by recognizing their sequences or/and RNA secondary structures through RNA-binding motifs. For example, the AUF1 protein recognizes RNAs through a signature motif composed of 29–39 nt with high A and U contents and a secondary structure specific to the RNAs (6). Binding of RBPs with RNA targets can also be regulated through competition with other RBPs and non-coding RNAs (7,8). RBPs may influence the global coordination of gene expression by organizing nascent groups of RNAs into downstream chains of the post-transcriptional modification process, through what is known as the ‘RNA-operon’ theory (9). RBPs have been implicated in various types of human diseases (1,10–13). For instance, the RBP Musashi1 was found to be related to many cancer types, including those of the breast, colon, medulloblastoma and glioblastoma, as well as to neurogenesis and neurodegenerative diseases (13). In addition, lack of Fragile X mental retardation protein (FMRP) results in a deficiency in human cognition and premature ovarian insufficiency (14) and the FUS, EWSR1, and TAF15 (FET) protein family is responsible for RNA editing and plays important roles in many diseases (15,16). In summary, studying RNA-protein interactions is necessary to achieve a systematic understanding of transcription, translation and other biological processes.

CLIP (cross-linking immunoprecipitation) is a molecular biology technology that employs ultraviolet (UV) cross-linking and immunoprecipitation in order to identify RBP–RNA interactions (17,18). The advantage of CLIP lies in allowing identification of interactions within cells (where the crosslinking occurs) versus interactions that might occur after cells are lysed. CLIP increases the confidence that observed interactions are physiologically relevant and can better justify identification of candidates for experimental validation. In early reports, CLIPed cDNAs were sequenced in a low-throughput manner that yielded a few hundred sequence reads. Recently, next-generation sequencing (NGS) techniques have been applied to globally analyzing transcriptional and post-transcriptional regulation, including mRNA sequencing (19), alternative splicing (20) and miRNA profiling (21). The combination of CLIP with NGS technology has greatly improved our ability to study RBP–RNA interactions on the genome scale (22). While earlier genome-wide CLIP studies focused more on the binding of RBP to mRNAs, recent studies have implicated a wide range of regulatory functions of RBP binding sites in long noncoding RNA (lncRNA) (23), circular RNA (24) and mitochondrial RNA (25).

In this study, we first review the general procedure and then compare current genome-wide CLIP technologies. Next, we discuss the major experimental design and bioinformatics analysis considerations. Finally, we provide an overview of the current analysis software and databases for genome-wide CLIP data.

### Current genome-wide CLIP technologies

**Figure 1.**
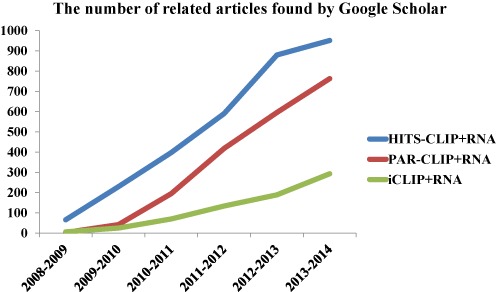
Number of related scientific articles found by Google Scholar by searching for each of the key terms in the given year interval. Since ‘iCLIP’ could have many other meanings, it is searched together with ‘CLIP-Seq’.

There are three major technologies for genome-wide CLIP experiments: (i) HITS-CLIP (high-throughput sequencing of RNA isolated by crosslinking immunoprecipitation) (22,26), which is the first version of genome-wide CLIP-Seq technology; (ii) Photoactivatable-Ribonucleoside-Enhanced Crosslinking and Immunoprecipitation (PAR-CLIP) (27), which improved the signal-to-noise ratio of the characteristic mutations observed in sequencing data by use of nucleoside analog; and (iii) Individual-nucleotide resolution CLIP (iCLIP) (28), which achieved a much higher efficiency in reverse-transcription compared with HITS-CLIP and PAR-CLIP. Throughout this text, we used genome-wide CLIP as a generic name for HITS-CLIP, PAR-CLIP and iCLIP. The field of RNA-regulation has seen rapid growth for all versions of genome-wide CLIP technology (Figure 1). In general, genome-wide CLIP technology involves cross-linking, partial RNA digestion, immunoprecipitation, reverse transcription and sequencing. The similarities and differences in the experimental procedures of these three CLIP methods are detailed below:

#### Cross-linking

The HITS-CLIP method exposes the processed biomaterials from cells or tissues to UV light to cross-link RNAs with bound RBPs (17). It was the first CLIP platform developed for the genome-wide identification of RBP binding sites. Although successful, it is limited by its low efficiency of UV-induced crosslinking, which makes it difficult to locate high-confidence binding sites. The PAR-CLIP method resolves this efficiency problem by incorporating photoreactive ribonucleoside analogs, such as 4-thiouridine (4-SU) and 6-thioguanosine (6-SG), into living cells in the culture system before the UV light treatment (27). Although ribonucleoside analogs improve the signal-to-noise ratio in PAR-CLIP data, treatment of living animals with these chemicals could be toxic. iCLIP employs a UV cross-linking strategy similar to HITS-CLIP.

#### Immunoprecipitation and enzymatic digestion

The immunoprecipitation step is similar for all HITS-CLIP, PAR-CLIP and iCLIP experiments. It generally involves bead preparation, cell lysis, partial RNA digestion immunoprecipitation, labeling and sodium dodecyl sulphate-polyacrylamide gel electrophoresis. The purified protein–RNA complexes are then treated by proteinase K. In the RNA digestion step, substantial bias could be introduced due to sequence specificity and amount of RNase being used. Less bias is expected with a low sequence-specificity RNase, like RNase I, and mild digestion strength. Importantly, recombinant ligase and proteinase K enzymes contain bacterial RNAs, mostly rRNAs. If the 3′ linker ligation is performed with free RNAs rather than with on-bead RNAs, these bacterial RNAs can also be cloned (29).

#### Reverse transcription

In HITS-CLIP experiments, the remaining cross-linked amino acid(s) are attached to the RNAs, which then become an obstacle for reverse transcription. The reverse transcriptase can read through these obstacles on cDNAs with a certain probability, but errors, reflected as mutations after sequencing, may be introduced on the cross-linking sites. In PAR-CLIP, chemical property changes as a result of nucleoside analog treatment (4SU for example) and UV light stimulus will induce a dA to dG mutation that can be detected in the sequencing data. These cross-linking induced mutations could serve as markers for RBP binding sites and are sometimes referred to as ‘characteristic mutations’. In HITS-CLIP experiments, the characteristic mutations could be substitutions, insertions, deletions or a combination of the above, depending on specific RBPs. For example, it has been shown that deletions are preferably induced in Argonaute (AGO) HITS-CLIP experiments (30). On the other hand, PAR-CLIP experiments induce a specific type of substitution depending on the nucleotide analog used: applying 4SU or 6SG leads to T→C or G→A substitutions, respectively (31).

In reverse transcription, a significant number of cDNAs will be truncated at the attached residues since the reverse transcriptase fails to read through these obstacles. These truncated cDNAs are normally not sequenced in HITS-CLIP and PAR-CLIP. The iCLIP procedure is designed to capture these truncation sites of cDNA fragments with high efficiency through replacement of the intermolecular ligation procedure with intramolecular circularization. Therefore, the 5′ ends of the sequencing reads, rather than characteristic mutations, are supposed to accurately mark the RBP targeting sites (28).

#### High-throughput sequencing

cDNA libraries can be subject to deep sequencing. Since the RNAs are sheared into short fragments of 20–100 bp, it was initially thought that single-end sequencing would usually be sufficient to cover whole cDNA fragments (32). However, some experiments require libraries of RNA fragments that are longer than those that could be covered by single-end sequencing, mainly due to dissimilar preferences in the library size selection step. Paired-end sequencing may be desirable in these cases so that whole cDNA fragments can be covered, because the lengths of RBP–RNA contact regions are comparable to the length of sequencing reads. Argonaute protein (AGO) is a key protein involved in RNAi that forms critical complexes with micro RNAs. AGO–RNA contact regions were estimated to be around 60 bp long (26). Therefore, exact coverage is important since identification of RBP binding sites usually requires a much higher resolution compared to ChIP-Seq experiments for transcription factors, whose resolution requirements are at least a few hundred base pairs (33).

## EXPERIMENTAL DESIGN AND BIOINFORMATICS ANALYSIS CONSIDERATIONS

The three variants of genome-wide CLIP experiments provide opportunities to understand RBP–RNA interactions on a genome-wide scale. There remain, however, many issues confronting experimental design, such as which CLIP method to use and how to conduct control and replicate experiments. The specific goal of the study should always be considered when making decisions regarding experimental design. For example, many earlier studies sought just to identify binding sites of the RBP of interest. More contemporary studies are concerned with RBP function such as splicing. Other recent studies are starting to venture into the realm of comparative analysis. Therefore, the genome-wide CLIP experiments should be designed differently to accommodate the specific goal of each study. Proper bioinformatics analysis should be carried out to best suit the choice of experimental procedure. In this section, we will discuss experimental design and bioinformatics analysis considerations following the natural order of how a genome-wide CLIP study is done.

### Choosing a CLIP method

The goal of a specific study is the primary consideration for choosing a CLIP method. Table 1 gives a brief summary of the advantages and disadvantages of the three genome-wide CLIP techniques that should be considered when choosing a CLIP version for the RBP under specific experimental conditions. Whether the experiment is to be done *in vivo* is one reason for favoring HITS-CLIP or iCLIP over PAR-CLIP, since the ribonucleoside analog treatment could be toxic. This is why HITS-CLIP and iCLIP have broad applications in cultured cells, animal tissues and plants. On the other hand, if the study wishes to reach a higher resolution at determining binding sites, PAR-CLIP or iCLIP should be favored. This is because PAR-CLIP has a much higher proportion of reads with characteristic mutations on cross-linking sites compared with HITS-CLIP and in iCLIP truncation sites can be directly used to accurately map interaction events. Thirdly, iCLIP is technically more challenging compared with HITS-CLIP and PAR-CLIP, which has probably limited its use. iCLIP requires the protein-bound RNA to be mildly digested by an endonuclease, which ensures the reads originating from truncated cDNA are long enough to be aligned. Therefore, a researcher needs to first experimentally determine the best condition to achieve an acceptable partial RNA digestion. In addition, iCLIP implements cDNA circularization and re-linearization steps. These steps require researchers to properly cut desired bands from polyacrylamide gels and carry out product elution and isolation. RNA obtained from CLIP techniques are generally in minute quantities. Extra manipulations on hardly-detectable cDNA will give an additional challenge to preparing an iCLIP sequencing library.

**Table 1.** Features of the three genome-wide CLIP platforms, as well as the major considerations for data analysis

	HITS-CLIP	PAR-CLIP	iCLIP
Ribonucleoside analog treatment	No	Yes (4-SU, 6-SG)	No
Cross-linking	UV light cross-linking	Ribonucleoside analog treatment and UV light cross-linking	UV light cross-linking
UV light wavelength	254 nm	365nm	254 nm
Adaptor ligations	Inter(molecular)/Inter	Inter/Inter	Inter/Intra
Diagnostic sites	No definite type of mutations	T→C or G→A	Pattern of cDNA truncations
PCR duplicates	Estimated by similarity in read sequence and alignment positions	Estimated by similarity in read sequence and alignment positions	Found by random barcodes
Advantages	Broad applications (from cultured cells, animal tissues and plants)	Enhanced UV-crosslinking efficiency; high signal-to-noise ratio at determining true binding sites	Broad applications; high signal-to-noise ratio at determining true binding sites
Disadvantages	Low characteristic mutation ratios	Potential toxicity of ribonucleoside analogs fed to cells	Technically more challenging

### Replicates

In RNA-Seq experiments, it has been shown that increasing the number of biological replicates consistently improves expression level quantifications and increases the statistical power to detect differentially expressed genes (34). It has become routine for most RNA-Seq experiments to have replicates to improve data quality and reproducibility. For genome-wide CLIP experiments, there is as yet no rigorous study on how the replicates affect the experimental results. Many genome-wide CLIP studies are based on a very limited number of replicated experiments, and replicates are often pooled during analysis (15,35). We examined 10 genome-wide CLIP studies published between 2009 and 2014 (15,26,36–43). We found that most experiments conducted 1–5 replicates per RBP under each treatment (Table 2) and most of these studies pooled the reads from replicates for the data analysis. The number of replicates that should be obtained depends on many factors, including the goal of the experiments, the variations of experiments, the sequencing depth and also the binding patterns of specific RBPs. For example, if the goal of the study is to conduct a comparative analysis between genome-wide CLIP conditions, then the quantification of within- versus between-group variation is very important and replicates will be of great value. A decision on the number of replicates to conduct can also take into consideration previously published studies for the experimental variations and binding patterns.

**Table 2.** Sequencing reads statistics for some genome-wide CLIP studies

Experiment	# Total sequencing reads (million by default)	# Unique sequencing reads (million by default)	# Uniquely mapped reads (million by default)	# Replicate	Method to handle replicates	Year	Citation
HITS-CLIP	26 (all replicates combined)	∼1.8 of all mapped reads	Unclear whether mapping allows non-unique alignment	5	Biologic complexity	2009	(26)
PAR-CLIP	4.1–33 (all replicates combined)	0.65–7.0	20–70% of sequencing reads after adaptor removal	1–7	Pooled	2010	(38)
iCLIP	6.5 (all replicates combined)	0.6 out of 4.2M uniquely mapped reads	4.2	3	Pooled	2010	(39)
PAR-CLIP	22–24 (all replicates combined)	Not reported	2.6–4.1	2	Pooled	2011	(15)
iCLIP	113 (all replicates combined)	33 out of 43M uniquely mapped reads	43	3	Focus on binding sites reproduced in all replicates	2012	(40)
HITS-CLIP	36–37 (second replicate)	0.95–1.5 out of 11M–15M uniquely mapped reads	11–15	2	Analyze the second replicate	2012	(36)
PAR-CLIP	60 (all replicates combined)	1.1	0.32	4	Pooled	2013	(37)
HITS-CLIP	72	0.35	0.22 out of 0.35M unique sequencing reads	1	NA	2014	(42)
HITS-CLIP	250–340 (each protein)	0.87–2.3	Not reported	4–5	Pooled	2014	(43)
iCLIP	169–433 (all replicates combined)	0.16–9.6 out of all mapped reads	12–48%	2	Pooled	2015	(41)

With respect to bioinformatics analysis, it is undesirable to pool the replicates. As each replicate could have a different sequencing depth, pooling will tend to down-weigh the replicates with less-sequenced reads. Moreover, the variation information on binding intensity at each binding site is lost after pooling. A measurement called biologic complexity (BC) has been applied to identifying RBP binding sites using replicates (26). Other than BC, PARma is the only algorithm that can consider replicates in its statistical algorithm (44). The DESeq package implements a statistical model that can incorporate replicate information to call differentially expressed regions (45). It was originally proposed for ChIP-Seq and RNA-Seq data, but could be adapted to CLIP-Seq studies where replicates are available (46). However, more advanced statistical approaches are also needed to address specific data features from CLIP-Seq experiments to better analyze such data with replicates more efficiently. In summary, no rigorous and comprehensive study has been conducted to investigate the effects of the number of replicates on statistical power and the accuracy of binding site detection for genome-wide CLIP experiments. Future studies and the development of bioinformatics tools for analyzing such experiments with replicates would improve the experimental design and data analysis.

### Control experiments

Most recently published genome-wide CLIP studies did not use background control experiments for identification of binding sites. Accordingly, few analysis approaches could process the sequencing data with both genome-wide CLIP and control conditions, with the exception of Piranha (47) in regression mode and dCLIP (48). Since genome-wide CLIP experiments involve stringent washes, such experiments without controls can still identify high-confident RBP binding sites. However, generating control experiments for CLIP studies would improve the accuracy and interpretation of the results. First, the ranking of identified binding sites from analyzing genome-wide CLIP data is usually biased toward abundantly expressed genes. If the CLIP cluster binding intensities are not normalized by control experiments, some clusters with high apparent binding strength could simply be intermediate-level-binding-strength sites on highly expressed RNA transcripts. Therefore, having background control experiments could help reduce such bias. Secondly, background RNA-Seq experiments could also help to identify SNPs in cell lines or tissue samples, as previously mentioned. In addition, if the study's goal is to understand RBP functions such as splicing, conducting an RNA-Seq experiment will help to discern which sites are functionally relevant. Konig *et* *al*. suggested a few ways to conduct background experiments (28) for iCLIP experiments, such as a no-antibody sample, non-crosslinked cells or immunoprecipitation from a knockout condition. Liu *et al*. experimentally showed that using input RNA or RNA-Seq in an experiment is also a good control (49). Again, the type of control experiment to conduct can vary and the choice depends on the specific goal of the study.

### Sequencing depth

Sequencing depth is a measure of the number of reads that are sequenced in one experiment. There is no consensus on the required sequencing depth for genome-wide CLIP experiments. We selected a few representative genome-wide CLIP studies as examples and summarized the summary sequencing statistics in each study (Table 2). The summary shows big variations in the total number of reads used in different studies, ranging from <10 million reads to more than 300 million reads for one experiment. The early studies generated low numbers of reads, while more recent studies generated much deeper reads for an RBP under one treatment. Due to the generally limited complexities of the cDNA libraries, very deep sequencing may not necessarily capture more unique events of RBP–RNA interactions for HITS-CLIP and PAR-CLIP experiments. However, this seems not to be the case for some iCLIP studies. The library complexities vary greatly for different CLIP experiments depending on many factors (50), such as how many binding sites the RBP under investigation truly binds. If the RBP has very specific binding sites, the expected library complexity would be small. Overall, the type of genome-wide CLIP experiment, the cost of sequencing and the number of true binding sites of the RBP should all be considered in determining the proper sequencing depth for the genome-wide CLIP experiments. Readers may refer to another review that thoroughly discusses the matter of sequencing depth in genomics studies (51).

### Mapping

Aligning the reads to a genome or transcriptome is the first step in genome-wide CLIP analysis. Mapping to a genome is usually chosen since there are sometimes many genome-wide CLIP clusters that locate within-reference gene introns. Mapping to a transcriptome or to both genome and transcriptome would be a good choice if the focus of the study is on detecting RBP binding sites on mature RNAs that have already been spliced. Table 3 lists commonly-used alignment software (52–56) for genome-wide CLIP datasets (26,44,47,57–62). In general, an aligner such as Gsnap that can handle short deletions and spliced-mapping will be a good choice. Gsnap is preferred by the CLIPper software (61) and it scored high in a systematic comparison of RNA aligners (63).

**Table 3.** Mapping software used in genome-wide CLIP analysis

Aligner	Title/Citation	Example studies
Bowtie	Ultrafast and memory-efficient alignment of short DNA sequences to the human genome (52)	(44,59–60)
Novoalign	http://www.novocraft.com/main/index.php	(57,58)
BLAT	BLAT—the BLAST-like alignment tool (53)	(26)
Gsnap	Fast and SNP-tolerant detection of complex variants and splicing in short reads (54)	(61)
BWA	Fast and accurate short-read alignment with Burrows–Wheeler transform (55)	(62)
RMAP	Updates to the RMAP short-read mapping software (56)	(47)

Another issue to consider is whether rRNAs, tRNAs and other types of repetitive sequences are of interest or should be removed by screening them from the pool. If they are not of interest, mapping to a pre-masked genome or removing rRNAs at the experimental stage using kits like Ribo-Zero may be more efficient. But this may not be the case with experiments that are conducted to make a comparative analysis, where 18S rRNAs can be used as a control invariant gene (64) Also, it is common practice for genome-wide CLIP data mapping to discard reads that can be mapped to multiple locations (15,57–58). However, some RBPs may have real binding sites in genes that have multiple copies in the genome. In such cases, discarding non-uniquely mapped reads will result in the loss of some true binding sites.

### PCR duplicates

Since genome-wide CLIP experiments involve polymerase chain reaction (PCR) amplification from cDNA libraries with limited complexities, removal of PCR duplicates amplified from common unique cDNA fragments is an important step. After duplicate removal, the size of the sequencing data usually drops dramatically (Table 2). There are a few ways to define PCR duplicates in genome-wide CLIP data. (i) Introducing random barcodes into the cDNA adaptor. This approach has been primarily applied to iCLIP experiments and has made it relatively easy to define PCR artifacts from the iCLIP data. Barcoding can give the clearest answer to whether a sequencing read is a PCR duplicate, and in fact it can also be applied to HITS-CLIP and PAR-CLIP, though this is not commonly done yet. PIPE-CLIP (65) has a bioinformatics procedure that can remove PCR duplicates according to barcodes for genome-wide CLIP data of all three sorts. (ii) For HITS-CLIP and PAR-CLIP, earlier studies defined PCR duplicates as sequencing reads having the same aligned genomic starting sites and duplicates were collapsed to a single sequencing tag (30). This may be too conservative, which usually leads to a collapsed sequencing read dataset that is <1/10 of its original size. (iii) Another popular approach adopted in many studies (48,66–67) is to define reads that have exactly the same mapping coordinates as PCR duplicates. (iv) Alternatively, it is also possible to define PCR duplicates as those having the same nucleotide sequence. Unfortunately, to our knowledge, there hasn't been any strict comparison reported in the literature to help select the best approach from (ii)–(iv) for HITS-CLIP and PAR-CLIP, and the scenario is even more complicated for paired-end sequencing reads. One consideration to choose among approaches (ii)–(iv) is the number of reads left after duplicate removal. If this step is too stringent, too few reads may be left for downstream analysis.

### Intron-locating clusters and spliced-mapping reads

Most genome-wide CLIP experiments do not distinguish nucleic RNAs from cytoplasmic RNAs because the RNA is obtained from whole cells. Since libraries could contain cDNAs converted from nascent pre-mRNAs, it is possible that a significant portion of CLIP reads will be mapped to reference gene introns. For example, in a few published studies, the proportions of intron-locating reads or CLIP clusters could be as low as 15% but also as high as 90% (68–70,36). This proportion depends on both the compartment of the cell that is being investigated and the property of the RBP under investigation. For example, Chu *et al*. found through PAR-CLIP that nucleic AGO2 preferentially binds intron regions while cytoplasmic AGO2 mainly binds 3′ UTR regions (71).

On the other hand, there are varying amounts of cDNAs generated from mature mRNAs in the libraries. Therefore, some of the sequencing reads could be mapped across splicing junctions. As a result, it is sometimes important to use an aligner that can handle splice junction mapping, or alternatively, to map the sequencing reads to the transcriptome in addition to the reference genome. However, usually fewer than 5% of all CLIP reads are mapped across splice junctions, due to two possible reasons: (i) only a small fraction of RBP binding sites are close to or on top of splice junctions or (ii) current aligners are not very efficient in mapping reads across splice junctions. CLIPZ (72) and PARma (44) are able to internally handle CLIP clusters that span junctions, while other pipelines such as dCLIP (48) must be fed externally with mapping data on both the genome and transcriptome in order to be splice-junction-aware.

### Peak-calling

Several statistical algorithms have been developed for peak-calling from genome-wide CLIP seq data and an overview of these algorithms will be detailed in the next section. The read counts are usually the primary measure for peak-calling from most algorithms and some statistical approaches were used to utilize the spatial patterns of the mapped reads. In addition, the characteristic mutations induced by cross-linking procedures have also been utilized to improve peak calling algorithms.

#### Characteristic mutations

In HITS-CLIP and PAR-CLIP, the cross-linking procedure induces mismatches in the final sequencing data, which could be used to pinpoint the location of RBP target sites at single-base-pair resolution and have been used to improve the binding target identifications. However, the proportion of sequencing reads with characteristic mutations varies greatly from 20%-80% for PAR-CLIP data (15,27,73,37). For HITS-CLIP data, the proportion is only around 10% (35) and even as small as <1% in one case (46). Another recent study (74) analyzed data from 20 genome-wide CLIP studies and found similar results. In addition, mutant bases are usually sparsely spread within CLIP clusters, normally leading to small ratios of mutant tags out of total tags on the exact cross-linking sites. Low mutant tag ratios in some experiments could be problematic for bioinformatics pipelines for analyzing HITS-CLIP and PAR-CLIP data that utilize mutation ratios, such as MiClip (75) and wavClusteR (31). On the other hand, there may be a small number of bases covered by CLIP clusters that show close to 100% mutant rates, which are likely SNPs in the cell lines or tissue samples instead of true RBP binding sites. To address these issues involving mutations, wavClusteR (31) introduces a parameter that effectively discards bases with mutation rates higher than a user-defined cutoff. Other ways to solve this problem include conducting control RNA-Seq experiments to detect SNPs or comparing results to databases of known SNPs. These observations, in addition to the obscurity of true characteristic mutations for some HITS-CLIP data, suggest that although characteristic mutation can help pinpoint the binding site and increase peak calling accuracy in most cases, careful examination of mutations from genome-wide CLIP experiments, especially from HITS-CLIP data, are necessary.

#### Using Hidden Markov Models (HMM) in binding sites detection

Similar to ChIP-Seq data, genome-wide CLIP reads counts are correlated among neighboring genomic locations, a phenomenon called spatial dependency. This occurs because protein binding regions span a certain length that is longer than the binned unit for counting binding intensity in the bioinformatics pipeline. In ChIP-Seq or ChIP-chip data analysis, it has been recognized that incorporating spatial dependency can greatly improve performance in identifying protein–DNA binding sites. Computational algorithms that consider this effect have been developed previously (76,77). However, these methods cannot be directly applied to genome-wide CLIP due to the unique features associated with genome-wide CLIP data: these data (i) are strand-specific; (ii) can reach a near-single base pair resolution; and (iii) contain information on cross-linking-induced mutations, which serve as markers for RNA–protein binding sites. An Hidden Markov Model (HMM) is a statistical model that could be used to model the observations with spatial dependency. In genome-wide CLIP data, HMM models usually have three main characteristics: (i) each genomic location has an unobserved hidden state indicating whether this location is a binding site, (ii) the hidden states along the genomic locations follow a Markov process, i.e. the hidden state of each location depend on only the states of its immediate neighboring locations; (iii) given the unobserved state, the observed total read/tag counts and mutations counts are independent across genomic locations, i.e. the dependency is in the hidden states. This structure enables the HMM to incorporate the spatial dependency of the genome-wide CLIP data. Among the tools developed for CLIP data, dCLIP and MiClip used HMM while also accounting for the other special properties of the genome-wide CLIP data and they have been shown to improve the identification of RBP-binding targets or differential binding sites.

### Downstream analysis: motif, structure and function

Following peak-calling, downstream analysis will generally focus on characterization of RBP–RNA interaction sites.

#### Motif discovery

Some RBP recognition motifs have been previously identified. For example, YCAY elements were found to be the biochemically-defined binding site for NOVA (78,79). Another study identified a 29–39 nt long AUF1 motif that contained 79% As and Us (6). HOMER (80) and MEME (81) are two popular bioinformatics tools for searching for sequence motifs. Zagros is a software that uses both secondary structure and characteristic mutations to improve motif discovery in genome-wide CLIP data (74). On the experimental side, an *in vitro* assay called RNAcompete was developed to determine RBP binding motifs from a pool of a complete range of k-mers in a single binding reaction (82). Much useful information has been gained from RNAcompete experiments, but the *in vivo* binding properties of the RBPs may be different from *in vitro* experiments. Compared with protein-binding motifs on DNAs, RNA sequence motifs tend to have less well-defined nucleotide preferences on each base and have degenerate and repeating elements.

#### Secondary structure

Also different from DNA–protein interactions, some RBPs recognize their targets mainly through RNA secondary structures or are sensitive to structural context (83,84), though paradoxically the RNAcompete method seems to make the contradictory observation that the vast majority of RBPs do not require RNA structures for specific binding. For example, the FUS protein has been shown to bind AU-rich stem-loops but does not seem to recognize any sequence motif (15). Interestingly, it has also been found that certain RBPs recognize single-strand RNAs, so intramolecular structures formed by the double-strand part of RNAs could actually inhibit RBP binding (85). Many tools have been developed to predict the secondary structures of RNA, such as CapR (86), RNAcontext (87) and RNAfold (88).

#### Functional characterization

Finally, it is important to investigate functions of identified RBP binding sites after peak-calling, since physical bindings may not necessarily lead to phenotypic consequences. The above-mentioned motif and structure information could be utilized to predict functional binding sites, such as in mCarts (89). Other high-throughput datasets, such as RNA expression, altnernative splicing or even clinical data, may also be integrated with genome-wide CLIP data to reveal functions of RBP–RNA interaction events. For example, one recent study (90) identified 22 735 RBP–lncRNA regulatory relationships from >100 public genome-wide CLIP datasets.

## OVERVIEW OF ANALYSIS METHODS AND DATABASE SERVERS

**Figure 2.**
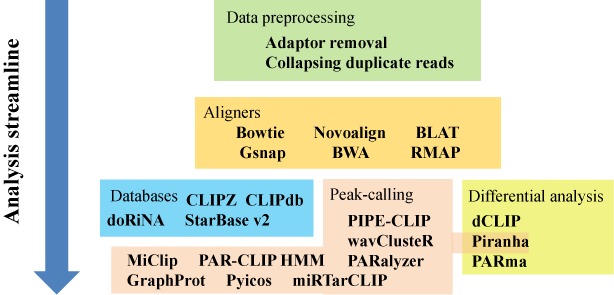
Summary of the analysis software, pipelines and databases for CLIP-Seq analysis mentioned in this review.

The previous section discussed some bioinformatics analysis approaches after high-throughput sequencing data has been aligned. In this section, we will give an overview of bioinformatics analysis software and databases for genome-wide CLIP experiments. Figure 2 shows a streamlined summary of genome-wide CLIP data analysis. Table 4 summarizes the major software programs, pipelines and databases to help readers choose the ones that best fit their purpose (65,31,44,46–48,50,72,75,91–99). We will discuss some of these in more detail in this section.

**Table 4.** Summary of genome-wide CLIP analysis software programs and databases

Software/Database	Type	Comment	Citation
CLIPZ	Database	Can carry out simple bioinformatics analysis	(72)
StarBase v2	Database	Contains CLASH datasets as well	(91,92)
doRiNA	Database	Focuses on miRNA biology	(93,94)
CLIPdb	Database	Contain uniformly identified binding sites of publicly available genome-wide CLIP datasets	(50)
PARalyzer	Software	Peak-finding algorithm for PAR-CLIP dataset only	(95)
Piranha	Software	Peak-finding and differential binding detection algorithm	(47)
dCLIP	Software	Differential binding detection algorithm	(48)
PIPE-CLIP	Software	Peak-finding algorithm	(65)
wavClusteR	Software	Peak-finding algorithm for PAR-CLIP dataset only	(31)
PARma	Software	Differential binding detection algorithm for AGO PAR-CLIP dataset only	(44)
MiClip	Software	Peak-finding algorithm wrapped as an R package	(75)
PAR-CLIP HMM	Software	Peak-finding algorithm employing Hidden Markov Model	(96)
GraphProt	Software	Peak-finding algorithm that can handle both RNAcompete and genome-wide CLIP data flexibly	(97)
Pyicos	Software	Peak-finding algorithm that can handle ChIP-Seq, genome-wide CLIP and RNA-Seq data flexibly	(98)
miRTarCLIP	Software	Peak-finding algorithm that employs a novel C to T reversion strategy in PAR-CLIP dataset analysis	(99)

### CLIPZ

CLIPZ is mainly a database for genome-wide CLIP datasets. There were 94 publicly-visible samples stored on CLIPZ as of April 2015. CLIPZ also provides simple bioinformatics analysis for stored samples. It first aligns the sequencing reads to genomes and transcriptomes, allowing alignments with more than one error (substitution, insertion or deletion). Then it generates clusters of sequencing reads and computes statistics like T->C substitutions for PAR-CLIP dataset. Finally, CLIPZ allows users to sort the clusters based on these computed features.

### StarBase v2

StarBase v2 is a database designed for decoding pan-cancer and interaction networks of RBPs, mRNAs and various types of non-coding RNAs from genome-wide CLIP datasets and CLASH datasets (100). As of April 2015, StarBase v2 contained 111 genome-wide CLIP datasets from 37 studies. StarBase v2 processes all the stored datasets and presents the analysis results through disparate portals such as miRNA–lncRNA interactions, miRNA–target interactions, protein–mRNA interactions and function predictions. The analysis conducted by StarBase v2 mostly relies on previously published software, such as PARalzyer for PAR-CLIP dataset analysis and TargetScan (101) and other similar pipelines for miRNA target site predictions.

### PARalyzer

PARalyzer is a popular peak-calling algorithm for PAR-CLIP datasets only. PARalyzer employs a non-parametric kernel-density estimation classifier to identify the RNA–RBP interaction sites using both total binding intensity information and T->C mutation information. It provides a dozen parameters, such as minimum number of reads and minimum number of conversions for a cluster, to help users filter the final results.

### Piranha

Piranha is mainly a peak-calling algorithm, but it also provides a way to detect differential binding across a range of conditions. All reads are binned and each bin represents a genomic interval. Piranha allows the users to flexibly choose an underlying model, including Poisson distribution and Negative Binomial distribution. It permits users to add additional covariates such as mutation data or transcript abundance data in a regression framework. This enables Piranha to incorporate mutation data in peak-finding or to conduct a differential binding analysis.

### dCLIP

dCLIP is designed specifically for identifying differential binding sites. The majority of the RBP binding sites between the two conditions should have roughly unchanged binding profiles, so dCLIP applies a MA-plot method to first normalize the two conditions. It uses a HMM to solve the common and differential binding sites. The HMM model incorporates the spatial dependency among neighboring locations to improve identification accuracy. Users can choose to input background transcript abundance profiling data as controls. dCLIP summarizes total tag count and mutant tag count data, as well as statistical inference results, into bedGraph and bed files that can be directly uploaded to Genome Browser for visualization.

### PIPE-CLIP

PIPE-CLIP is a Galaxy-based comprehensive online pipeline for genome-wide CLIP data analysis. It processes BAM files by filtering out reads that do not meet mismatched numbers and/or aligned read-length criteria and by removing PCR duplicates according to reads locations or sequences. Then it applies zero-truncated negative binomial regression to identify the enriched clusters and fits a binomial distribution to assess the significance of featured mutations/truncations. After that, enriched clusters with significant mutations/truncations are reported as binding sites.

### wavClusteR

wavClusteR is designed for identifying RBP peaks in a single PAR-CLIP experiment. It defines a mixture model where the first component indicates random substitutions, which are not induced by cross-linking and the second component indicates cross-linking-induced substitutions that serve as markers of RBP-protein binding sites. wavClusteR relies on the assumption that all types of non-experimentally-induced substitutions have approximately the same distribution as the first component, while only PAR-CLIP-induced T->C mutations exist in the second component of the mixture model. However, this may not be the case for tumor cell lines where the background mutation profiles are distinct for each type of substitution (102).

### MiClip

MiClip is an R package for identifying RBP binding sites using HITS-CLIP and PAR-CLIP datasets. It leverages the spatial dependency in sequencing tag data by using HMM and it also takes advantage of characteristic mutation counts to increase peak-calling accuracy. It is user-friendly and requires only that users feed in several parameters to optimize the performance of the algorithm. MiClip is freely available on CRAN (The Comprehensive R Archive Network).

### PARma

PARma is a tool for differential AGO PAR-CLIP data analysis. In PARma, a statistical model and a novel pattern discovery tool are iteratively applied to estimate probabilities and to assign the most probable miRNAs until convergence. The statistical model is composed of three independent parts that consider the T->C mutation frequencies as well as the properties of the nucleotide compositions at both ends of the sequencing reads. The PARma algorithm addresses several important issues in the data preprocessing step, such as the handling of spliced-mapping reads and consideration of experimental replicates. However, it can only be applied to differential AGO PAR-CLIP datasets.

## CONCLUSION AND DISCUSSION

In this review, we discussed the genome-wide CLIP technology from the perspectives of experimental design and bioinformatics analysis. The development of technology and bioinformatics in this field has greatly improved our capacity to study protein–RNA interactions and understand the functions of different RNA species in physiological and pathological process. There are several related technologies, such as CLASH and RIP-Seq, which may be complementary to genome-wide CLIP to study the function of RNAs. CLASH is short for cross-linking, ligation and sequencing of hybrids, which was invented for characterizing intramolecular and intermolecular RNA–RNA interactions (100). Recently, this technology was adapted to straightforwardly detect miRNA–mRNA pairs as chimeric reads in high-throughput sequencing data (103). Integrative analysis can be carried out that combines CLASH data that can directly capture reliable miRNA–mRNA interactions and genome-wide CLIP data that focuses more on detecting RBP–RNA interactions. RNA immunoprecipitation sequencing (RIP-Seq) can also complement genome-wide CLIP for identifying RBP–RNA interactions (104). RIP-Seq bears some similarity to genome-wide CLIP, but lacks high-stringency washes and crosslinking of RBP to RNAs, which leads to high background noise and mis-interpretations in the data analysis. For example, RIP-Seq identifies both direct and indirect RBP–RNA interactions, while genome-wide CLIP can accurately identify direct RBP–RNA association events (105). However, genome-wide CLIP is more technically challenging and also requires high-quality antibodies to work properly. Therefore the data from CLIP experiments and RIP-Seq experiments could be complementary in studying RBP–RNA bindings.

The genome-wide CLIP has accumulated extensive knowledge in both experimental procedures and how to process genome-wide CLIP data properly, but it requires fundamental improvements to reach its potential. First, more systematic studies on experimental design issues such as replicates, the use of background controls and the sequencing depth are greatly needed to improve the experimental efficiency, reduce systematic bias and increase the reproducibility of genome-wide CLIP experiments. Another direction for further study is to conduct genome-wide CLIP experiments of different proteins under different treatments simultaneously in an experimental system to methodically understand and model transcriptional events. The MOV10 and UPF1 proteins have recently been shown to bind in close proximity and interact directly (106), pointing to the importance of studying the coordination pattern of RBPs and its functional impact. A third future direction is to combine genome-wide CLIP with other types of data, including ChIP-Seq, RNA-Seq and proteomics data for integrative analysis. EZH2 was reported to bind lncRNAs (37), despite its chromatin-binding capability and its role in epigenetic regulation. This intriguing phenomenon suggests that ChIP-Seq data and genome-wide CLIP data can be analyzed together to reveal novel RNA-binding functions of well-characterized DNA-binding proteins. There are a lot of interesting discoveries yet to be made from mining genome-wide CLIP data. All of these efforts will help us better understand transcriptional regulation in biological systems.
